# Supplementary material for: Predicted IL-18/IL-18R Binding Improvement Through Protein Interface Modification with Computer-Aided Design
Source: Biomolecules. 2025 Sep 25;15(10):1360. doi: 10.3390/biom15101360 (PMC12564372; doi:10.3390/biom15101360)
Supplement: Supplementary file 1 [file biomolecules-15-01360-s001.zip › biomolecules-3848669-supplementary.pdf]

SUPPLEMENTARY MATERIALS

Predicted IL-18/IL-18R Binding Improvement through Protein Interface Modification with Computer-aided Design

Napat Prompat, Chariya Peeyatu, Jirakrit Saetang, Niran Roongsawang, Surasak Sangkhathat and Varomyalin Tipmanee*

Figure

- **Figure S1.** Decomposition binding free energy of each residue of IL-18. The key residues involved in the IL18 binding site I, II, and III were colored in yellow, blue, and pink respectively.
- **Figure S2.** Schematic representations of the interaction profiles between IL-18/IL-18Rα complex. Interfacial residues of IL-18 and IL-18Rα are shown in red and white licorice presentation, respectively.
- **Figure S3.** Per residue free energy contribution of the key residues in the binding site I and II of IL-18. The energy in kcal/mol was categorized into the electrostatic contribution (red dot), van der Waals contribution (blue dot), desolvation contribution (gray dot) and the total binding free energy (black line).
- **Figure S4.** Correlation between relative activities of IL-18 and the predicted binding free energies ($\Delta\Delta G_{binding}$) of IL-18/IL-18R complex obtained from FoldX's interaction energy calculation. ΔΔG value is expressed in kcal/mol. **Correlation is significant at the 0.01 level (p-value < 0.01).
- **Figure S5.** Correlation between relative activities of IL-18 and the predicted free energies of folding ($\Delta\Delta G_{folding\ of\ complex}$) of IL-18/IL-18R complex obtained from FoldX's relative free energy calculation. ΔΔG value is expressed in kcal/mol. **Correlation is significant at the 0.01 level (p-value < 0.01).
- **Figure S6.** Correlation between relative activities of IL-18 and the predicted free energies of folding ($\Delta\Delta G_{folding\ of\ isolated\ ligand}$) of IL-18 obtained from FoldX's relative free energy calculation. ΔΔG value is expressed in kcal/mol. *Correlation is significant at the 0.05 level (p-value < 0.05).
- **Figure S7.** 3D structure of IL-18 in complex with IL-18Rα. The selected candidate residues (E6, N111, K129 and R131) show in Licorice. The structure of human IL-18 (green) and IL-18Rα (grey).
- **Figure S8.** The superimposition of the average structure from last 90 ns of simulation of IL-18 wild-type (green) and its mutants (colors) in complex with IL-18Rβ (gray). Wild-type (green), E6M (orange), E6M-N111S-R131G (red), E6M-K129M-R131G (cyan) and E6M-N111S-K129M-R131G (purple). Red arrow indicated the amino acid residues involved in β8-β9 loop region of IL-18 mutants bind closer to the receptor (IL-18Rβ) compared to the wild-type.
- **Figure S9**. Per-residue RMSD (black line) and RMSF (red line) of IL-18 wild-type.
- **Figure S10**. Per-residue RMSD (black line) and RMSF (green line) E6K mutant.

- **Figure S11**. Per-residue RMSD (black line) and RMSF (pink line) of M33Q mutant.
- **Figure S12**. Per-residue RMSD (black line) and RMSF (blue line) of E6M mutant.
- **Figure S13**. Per-residue RMSD (black line) and RMSF (beige line) of E6M-N111S-R131G mutant.
- **Figure S14**. Per-residue RMSD (black line) and RMSF (yellow line) of E6M-K129M-R131G mutant.
- **Figure S15**. Per-residue RMSD (black line) and RMSF (purple line) of E6M-N111S-K129M-R131G mutant.

Table

- **Table S1.** Comparison of experimental activity and *in silico* predicted relative free energy (ΔΔG) in human IL-18.
- **Table S2.** Binding interactions between the E6M mutant/IL-18R interfaces at binding site I, II and III.
- **Table S3.** Binding interactions between the E6M+K129M+R131G mutant/IL-18R interfaces at binding site I, II and III.
- **Table S4.** Binding interactions between the E6M+N111S+R131G mutant/IL-18R interfaces at binding site I, II and III.
- **Table S5.** Binding interactions between the E6M+N111S+K129M+R131G mutant/IL-18R interfaces at binding site I, II and III.

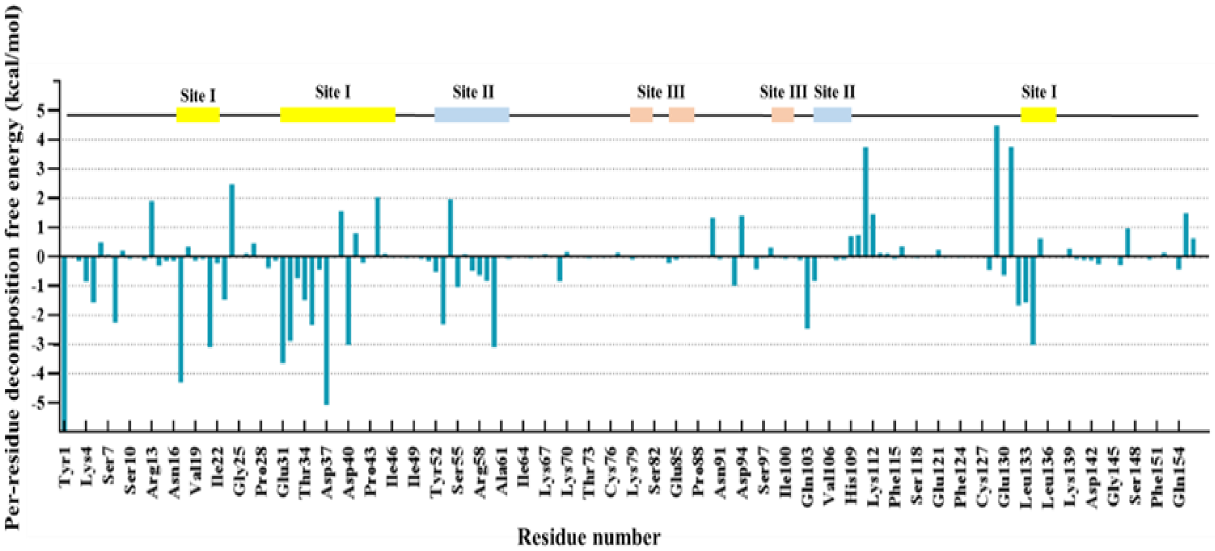

Figure S1. Decomposition binding free energy of each residue of IL-18. The key residues involved in the IL18 binding site I, II, and III were colored in yellow, blue, and pink respectively.

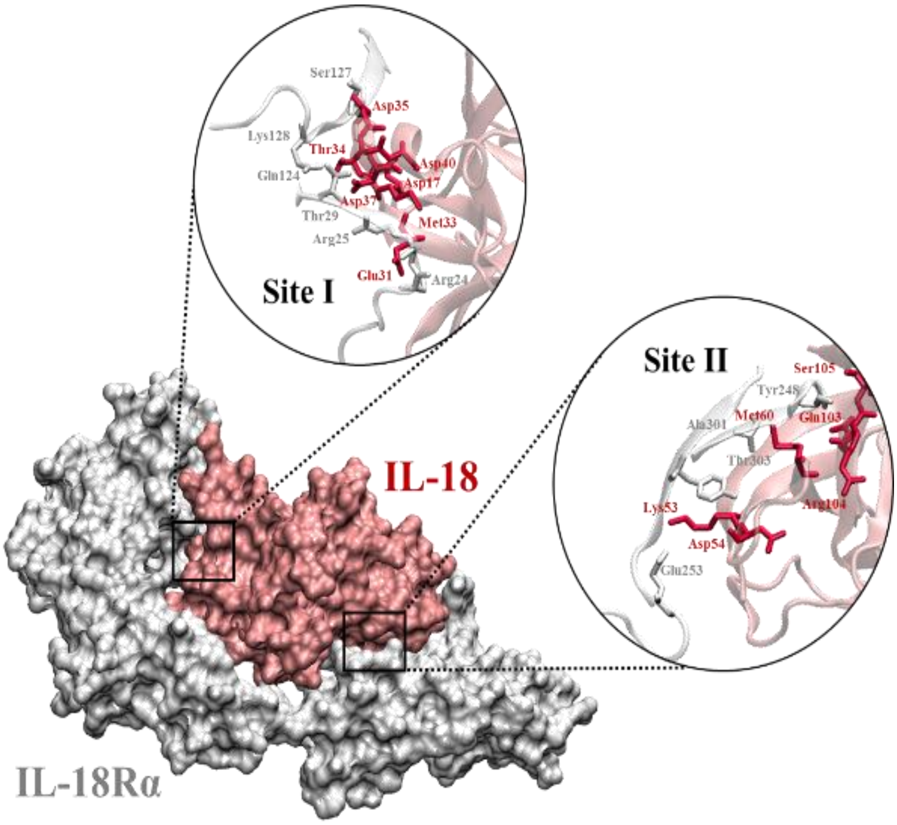

Figure S2. Schematic representations of the interaction profiles between IL-18/IL-18Rα complex. Interfacial residues of IL-18 and IL-18Rα are shown in red and white licorice presentation, respectively.

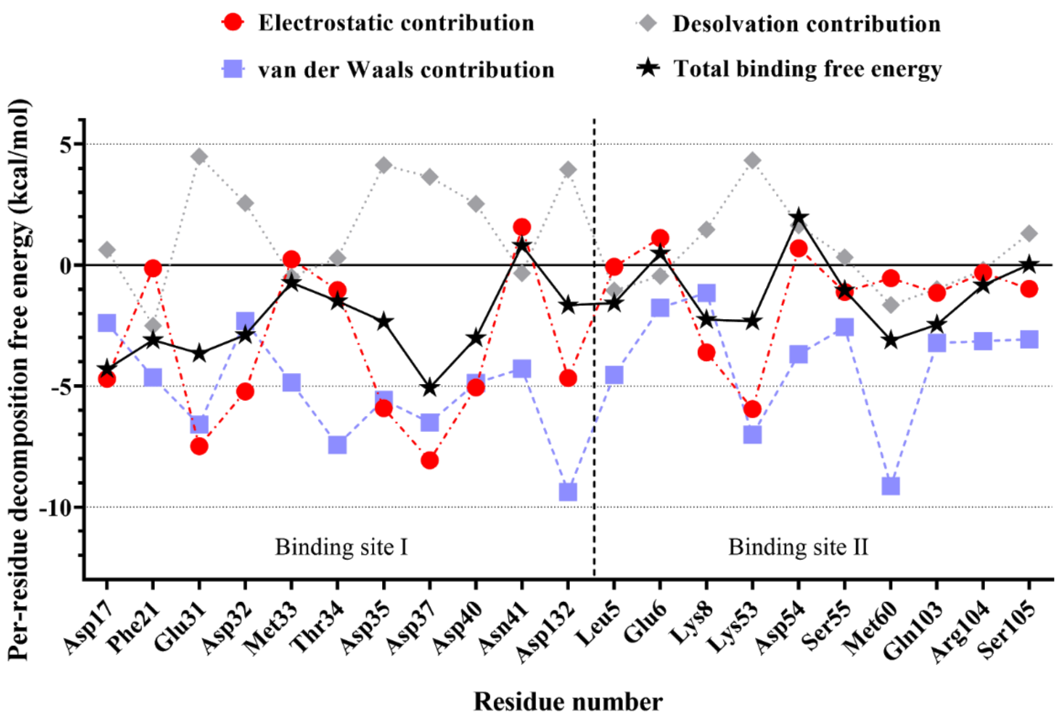

Figure S3. Per residue free energy contribution of the key residues in the binding site I and II of IL-18. The energy in kcal/mol was categorized into the electrostatic contribution (red dot), van der Waals contribution (blue dot), desolvation contribution (gray dot) and the total binding free energy (black line).

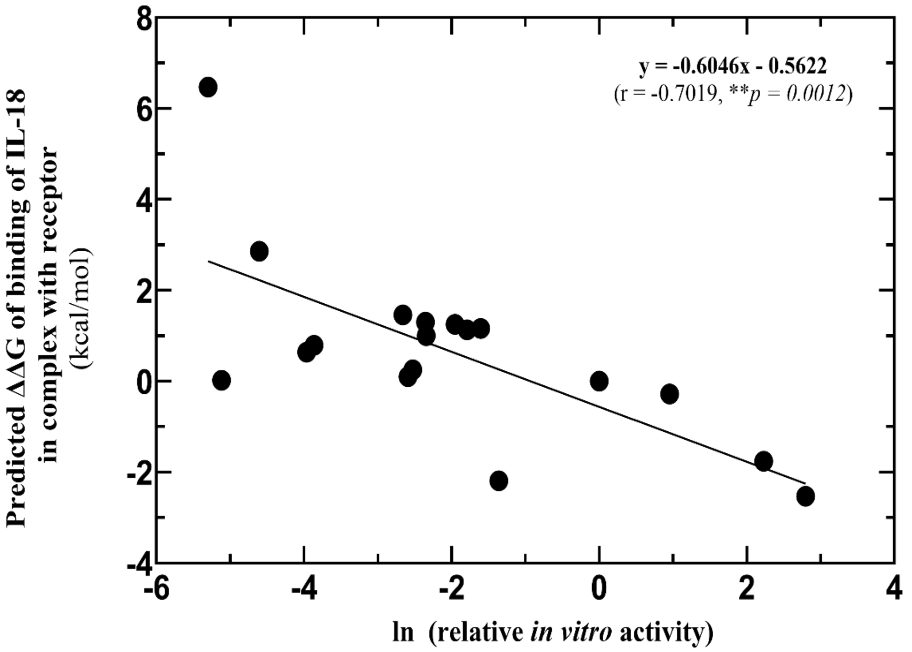

Figure S4. Correlation between relative activities of IL-18 and the predicted binding free energies ($\Delta\Delta G_{binding}$) of IL-18/IL-18R complex obtained from FoldX's interaction energy calculation. ΔΔG value is expressed in kcal/mol. **Correlation is significant at the 0.01 level (*p*-value < 0.01).

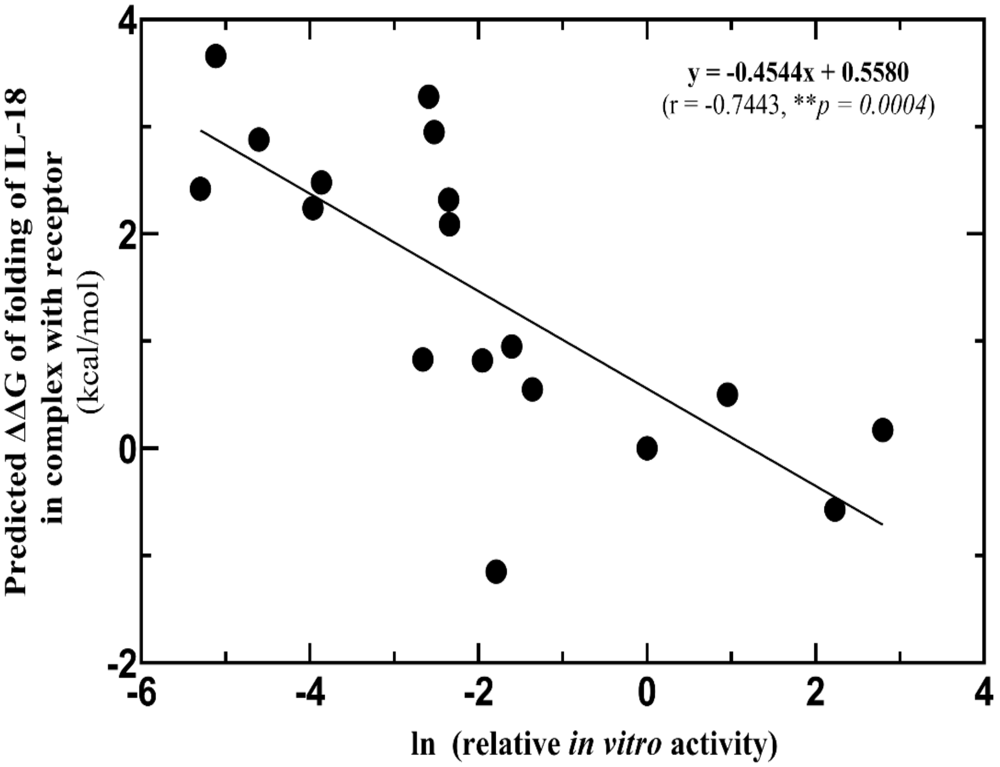

Figure S5. Correlation between relative activities of IL-18 and the predicted free energies of folding ($\Delta\Delta G_{folding\ of\ complex}$) of IL-18/IL-18R complex obtained from FoldX's relative free energy calculation. ΔΔG value is expressed in kcal/mol. **Correlation is significant at the 0.01 level (*p*-value < 0.01).

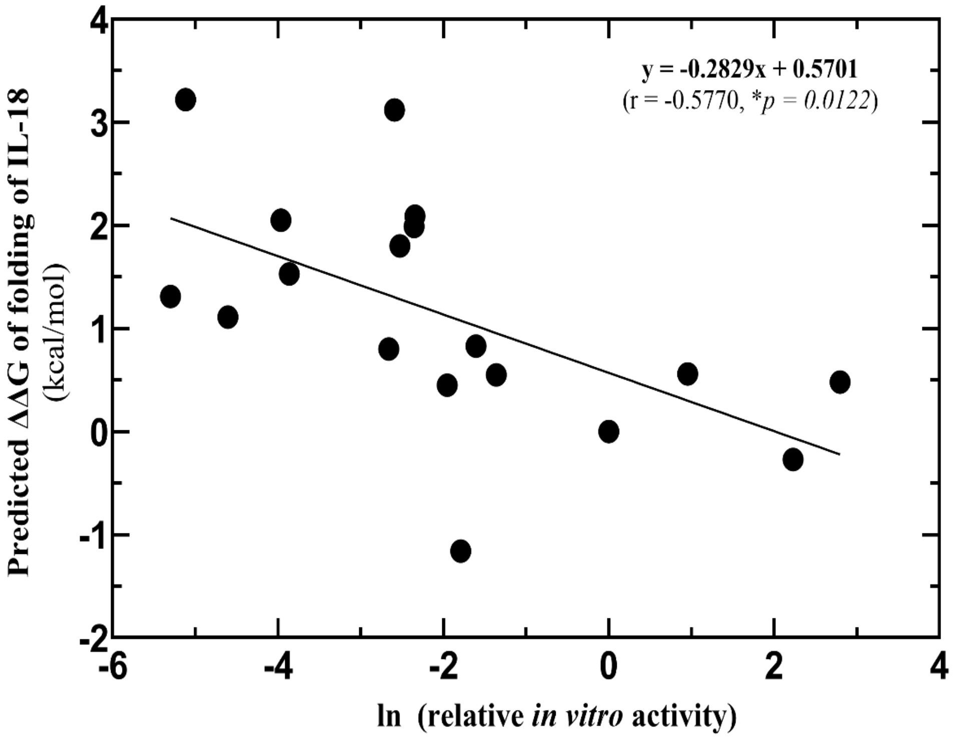

Figure S6. Correlation between relative activities of IL-18 and the predicted free energies of folding ($\Delta\Delta G_{\text{folding of isolated ligand}}$) of IL-18 obtained from FoldX's relative free energy calculation. ΔΔG value is expressed in kcal/mol. *Correlation is significant at the 0.05 level (p-value < 0.05).

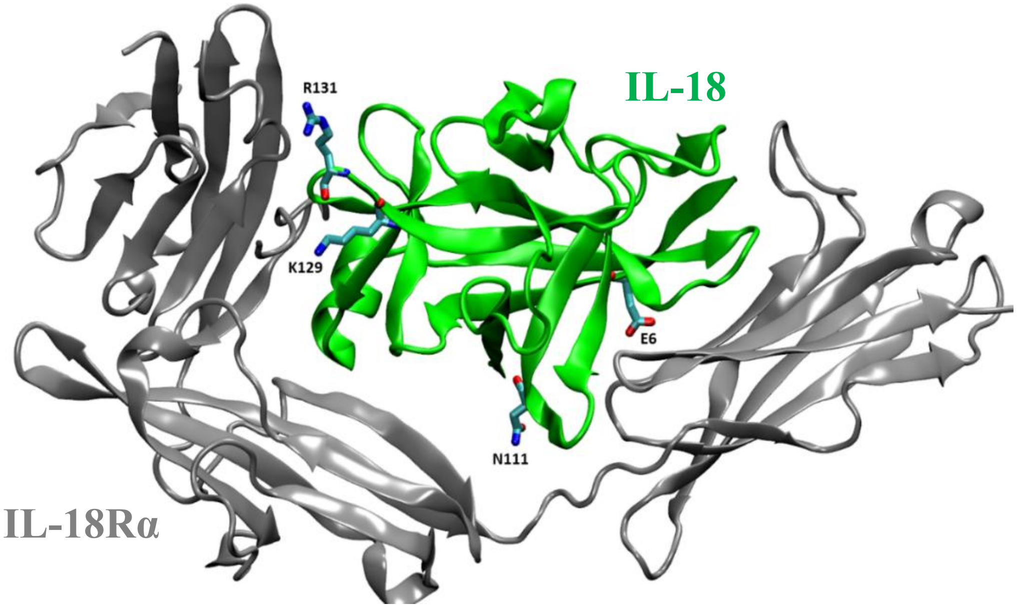

Figure S7. 3D structure of IL-18 in complex with IL-18Rα. The selected candidate residues (E6, N111, K129 and R131) show in Licorice. The structure of human IL-18 (green) and IL-18Rα (grey).

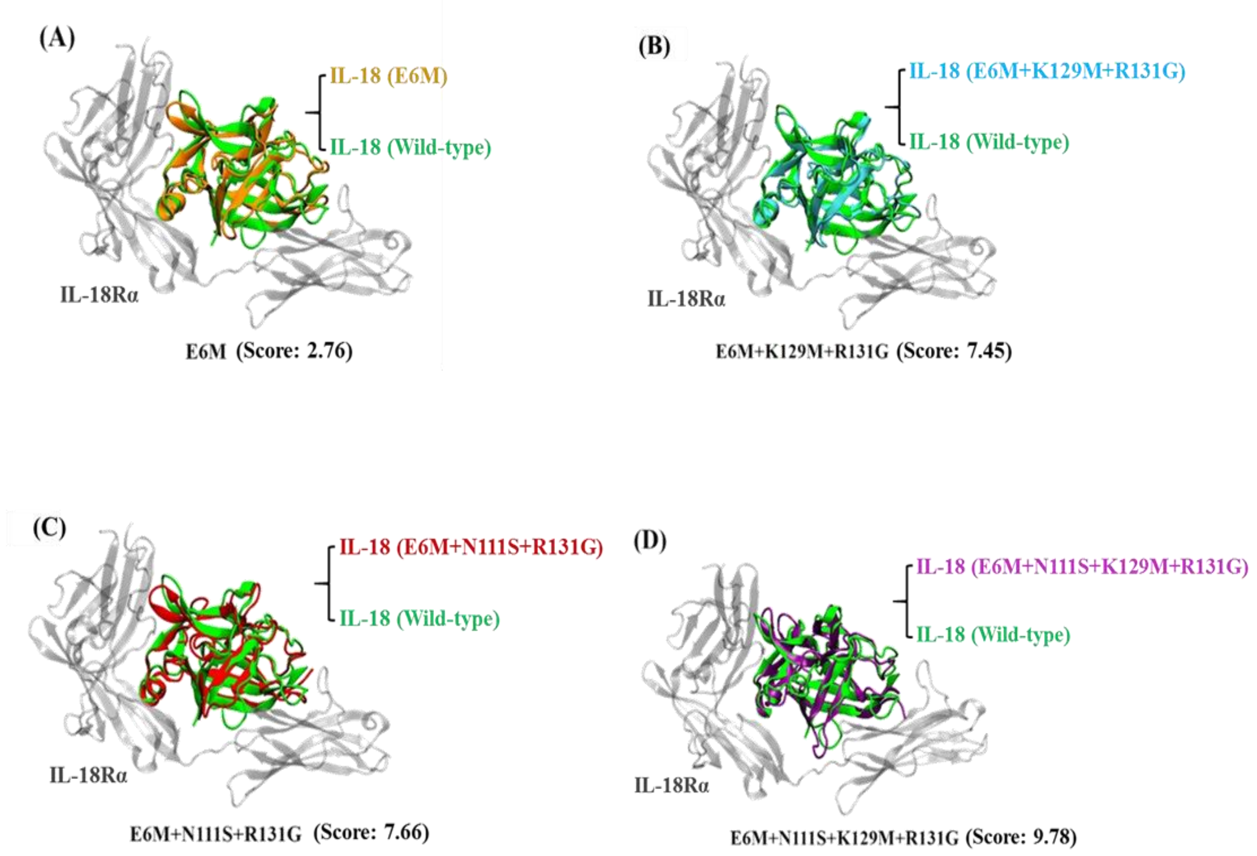

Figure S8. The superimposition of the average structure from last 90 ns of simulation of IL-18 wild-type (green) and its mutants (colors) in complex with IL-18Rβ (gray). Wild-type (green), E6M (orange), E6M-N111S-R131G (red), E6M-K129M-R131G (cyan) and E6M-N111S-K129M-R131G (purple). Red arrow indicated the amino acid residues involved in β8-β9 loop region of IL-18 mutants bind closer to the receptor (IL-18Rβ) compared to the wild-type.

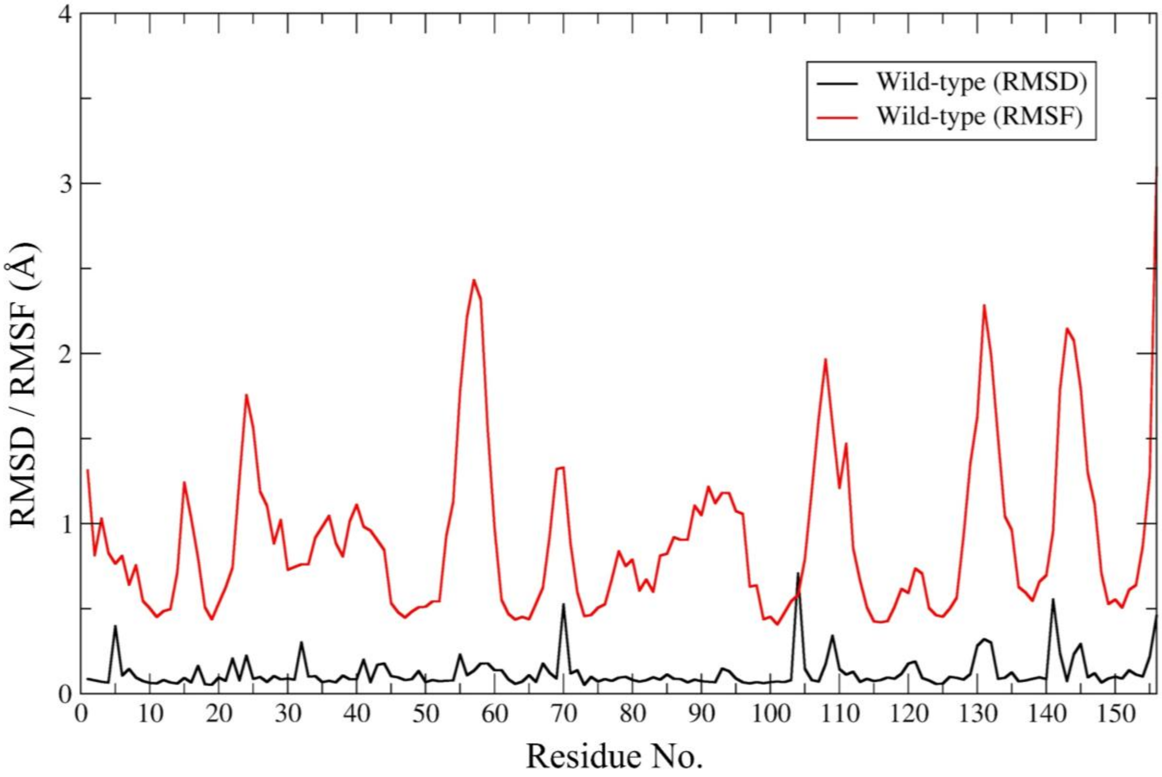

Figure S9. Per-residue RMSD (black line) and RMSF (red line) of IL-18 wild-type.

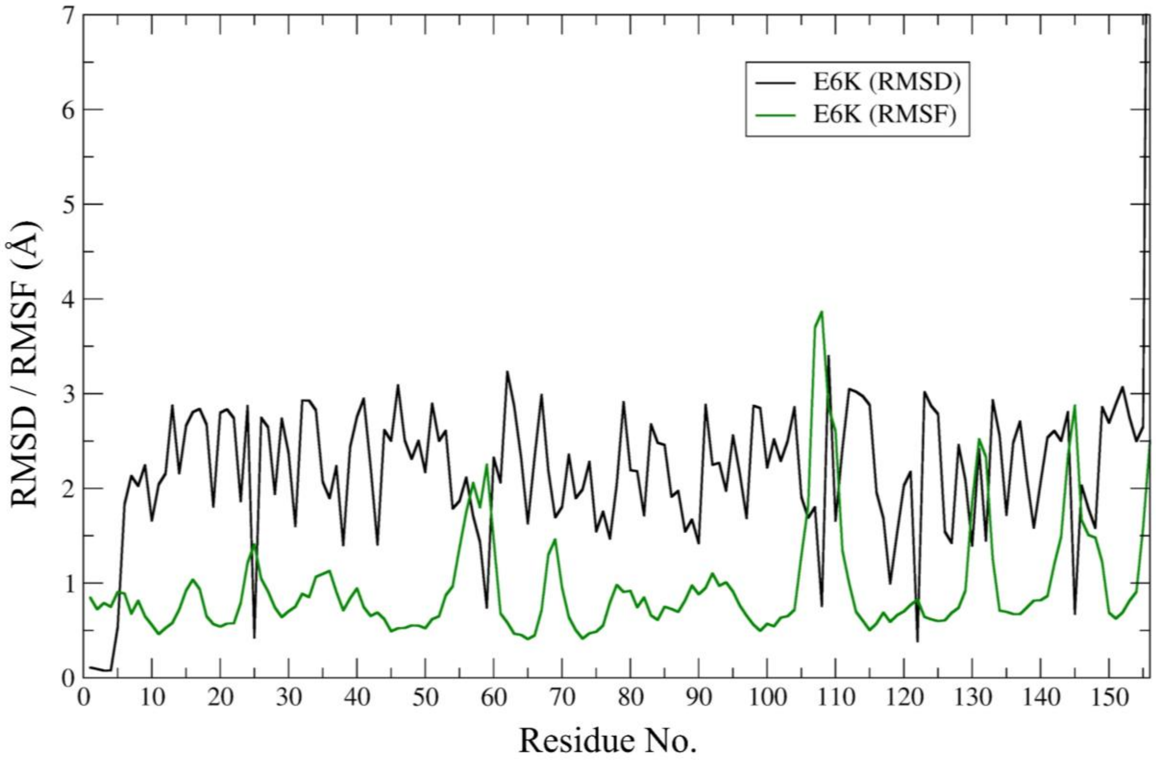

Figure S10. Per-residue RMSD (black line) and RMSF (green line) E6K mutant.

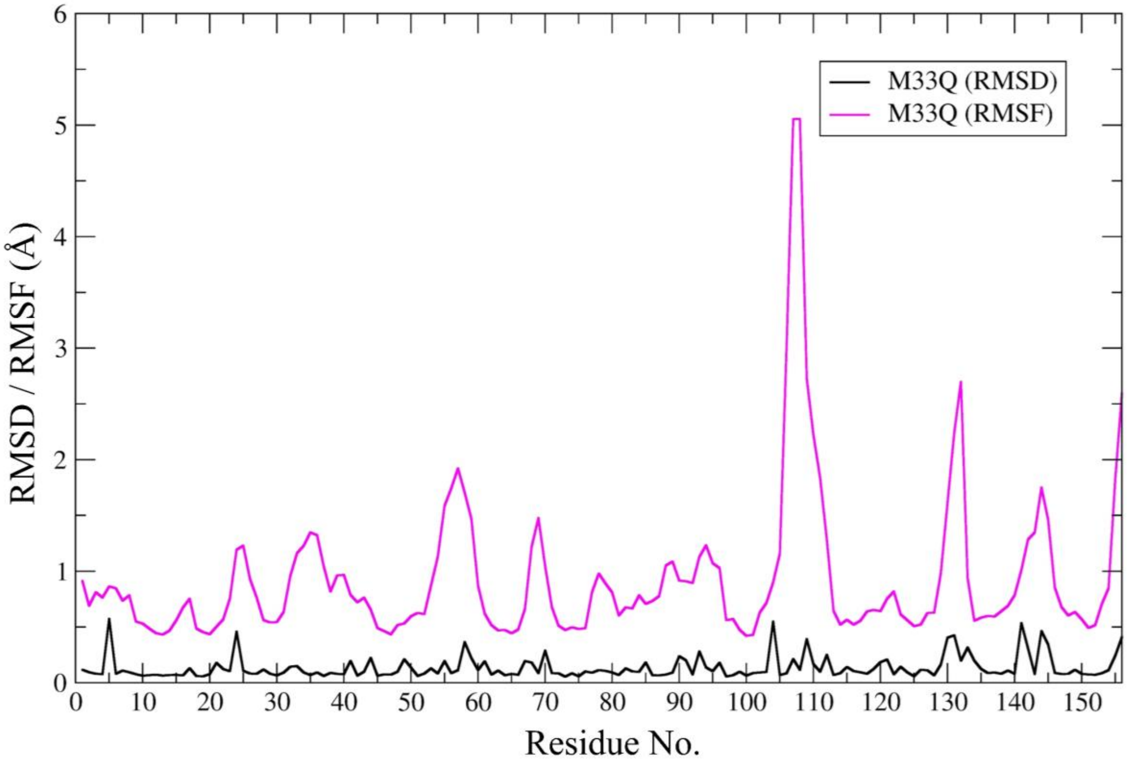

Figure S11. Per-residue RMSD (black line) and RMSF (pink line) of M33Q mutant.

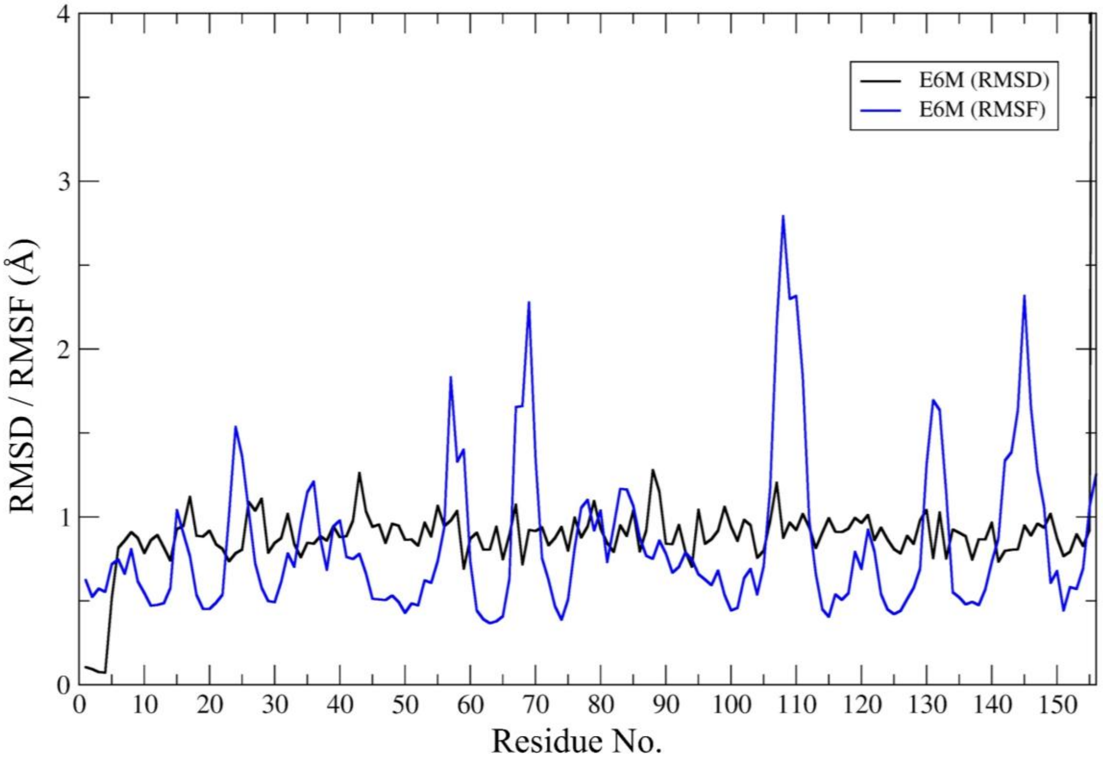

Figure S12. Per-residue RMSD (black line) and RMSF (blue line) of E6M mutant.

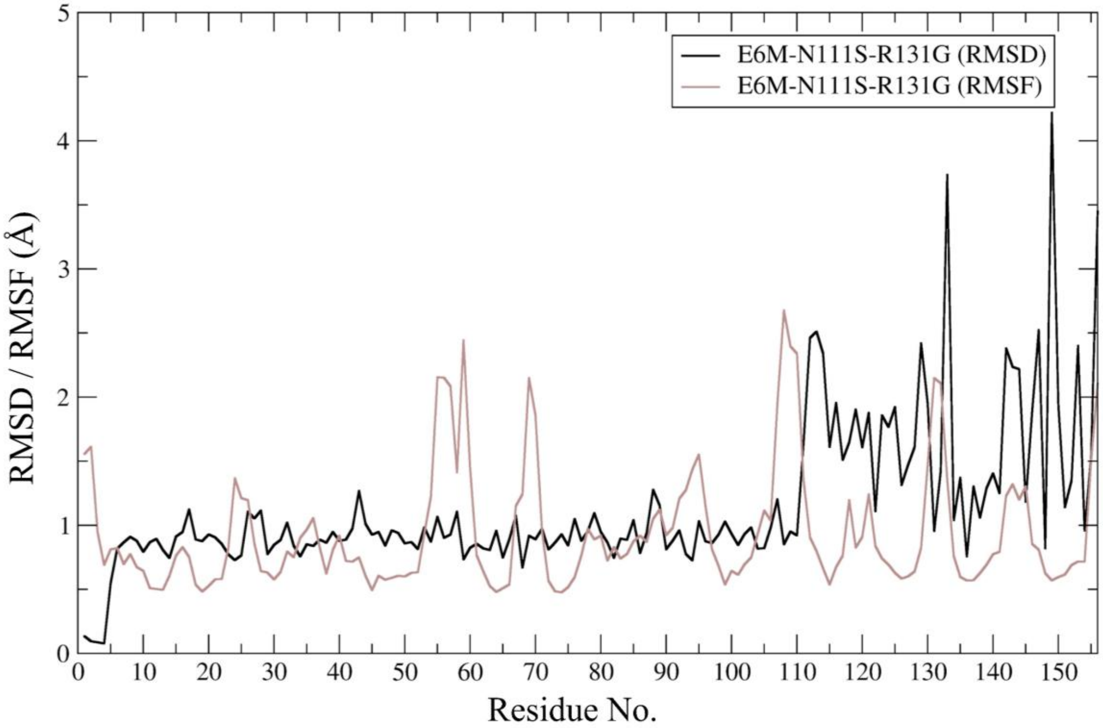

Figure S13. Per-residue RMSD (black line) and RMSF (beige line) of E6M-N111S-R131G mutant.

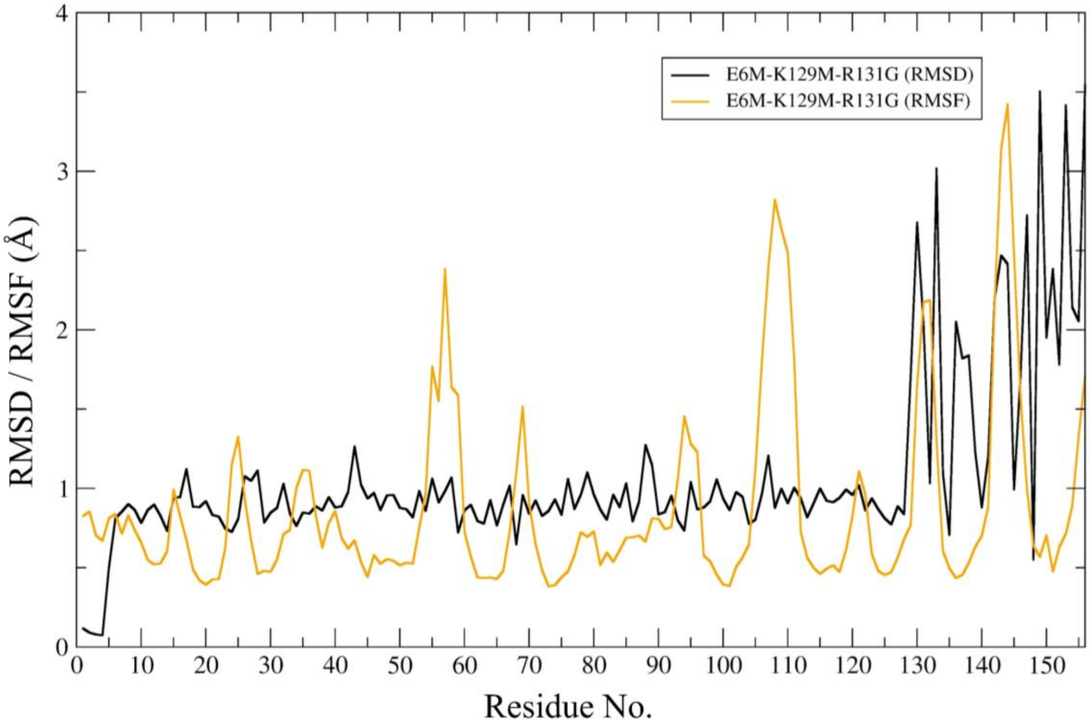

Figure S14. Per-residue RMSD (black line) and RMSF (yellow line) of E6M-K129M-R131G mutant.

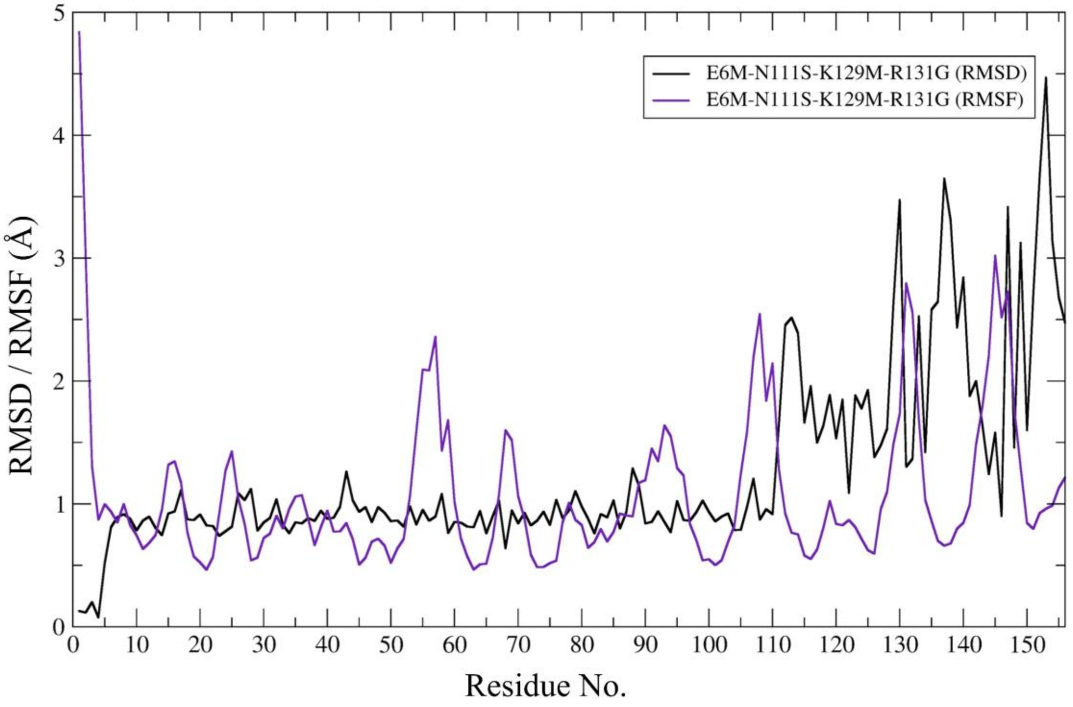

Figure S15. Per-residue RMSD (black line) and RMSF (purple line) of E6M-N111S-K129M-R131G mutant.

Table S1. Comparison of experimental activity and *in silico* predicted relative free energy (ΔΔG) in human IL-18.

Mutation	Relative activity [a]	ΔΔG binding of IL-18/IL-18R complex	ΔΔG folding of IL-18/IL-18R complex	ΔΔG folding of IL-18	Ref.
**Wild-type**	1	0	0	0	Saetang et al., 2016
**K4A**	0.07	1.46	0.83	0.80	Kato et al., 2003
**L5A**	0.01	2.86	2.88	1.11	Kato et al., 2003
**K8A**	0.095	1.3	2.32	1.99	Kato et al., 2003
**D17A**	0.21	1.16	0.95	0.83	Kato et al., 2003
**R13A**	0.08	0.25	2.95	1.80	Kato et al., 2003
**M33A**	0.006	0.02	3.66	3.22	Kato et al., 2003
**D35A**	0.005	6.47	2.42	1.31	Kato et al., 2003
**R58A**	0.019	0.64	2.24	2.05	Kato et al., 2003
**M60A**	0.021	0.79	2.48	1.53	Kato et al., 2003
**R104A**	0.075	0.1	3.28	3.12	Kato et al., 2003
**D132A**	0.142	1.25	0.82	0.45	Kato et al., 2003
**K79A**	0.096	1	2.09	2.09	Kato et al., 2003
**K84A**	0.257	-2.19	0.55	0.55	Kato et al., 2003
**D98A**	0.167	1.13	-1.15	-1.16	Kato et al., 2003
**E6A**	2.6	-0.28	0.50	0.56	Kato et al., 2003
**E6K**	9.3	-1.76	-0.57	-0.27	Saetang et al., 2016
**E6K+T63A**	16.4	-2.53	0.17	0.48	Saetang et al., 2016

[a] The biological activity of IL-18 was evaluated experimentally by IFN-γ induction assay in NK-92MI cells. Relative activities of mutants were determined by a ratio of a reported activity from mutant IL-18 to an activity from wild-type IL-18.

Table S2. Binding interactions between the E6M mutant/IL-18R interfaces at binding site I, II and III.

Binding site	IL-18 residue	IL-18R residue	Interaction
Site I	D17	K128	Electrostatic interaction
	D32	R25	Electrostatic interaction
	D40	H27	Electrostatic interaction
	D132	K39	Electrostatic interaction
Site II	K53	E263	Electrostatic interaction
Site III	D110	K313	Electrostatic interaction
	K112	E210	Electrostatic interaction
	K112	Y212	Cation-π interaction
	R147	E210	Electrostatic interaction

Table S3. Binding interactions between the E6M+K129M+R131G mutant/IL-18R interfaces at binding site I, II and III.

Binding site	IL-18 residue	IL-18R residue	Interaction
	D17	K128	Electrostatic interaction
	D32	R25	Electrostatic interaction
**Site I**	D40	H27	Electrostatic interaction
	D40	R123	Electrostatic interaction
	D132	K39	Electrostatic interaction
**Site II**	K53	E263	Electrostatic interaction
	H109	E210	Electrostatic interaction
	D110	K313	Electrostatic interaction
**Site III**	K112	E210	Electrostatic interaction
	K112	Y212	Cation-π interaction
	R147	E210	Electrostatic interaction

Table S4. Binding interactions between the E6M+N111S+R131G mutant/IL-18R interfaces at binding site I, II and III.

Binding site	IL-18 residue	IL-18R residue	Interaction
Site I	D17	K128	Electrostatic interaction
	D32	R25	Electrostatic interaction
	D40	H27	Electrostatic interaction
	D132	K39	Electrostatic interaction
Site II	K53	E263	Electrostatic interaction
Site III	H109	E210	Electrostatic interaction
	H109	Y212	π-π stacking / cation-π interaction
	D110	K313	Electrostatic interaction
	K112	E210	Electrostatic interaction
	K112	Y212	Cation-π interaction
	R147	E210	Electrostatic interaction

Table S5. Binding interactions between the E6M+N111S+K129M+R131G mutant/IL-18R interfaces at binding site I, II and III.

Binding site	IL-18 residue	IL-18R residue	Interaction
Site I	D17	K128	Electrostatic interaction
	D17	F202	Anion-π interaction
	D32	R25	Electrostatic interaction
	D37	R25	Electrostatic interaction
	D40	H27	Electrostatic interaction
	D40	R123	Electrostatic interaction
	D132	K39	Electrostatic interaction
Site II	K53	Y248	Cation-π interaction
Site III	H109	E210	Electrostatic interaction
	D110	E210	Electrostatic interaction
	K112	E210	Electrostatic interaction
	K112	Y212	Cation-π interaction
	D146	Y214	Anion-π interaction
